# SOX2, JAGGED1, β-Catenin, and Vitamin D Receptor Expression Patterns during Early Development and Innervation of the Human Inner Ear

**DOI:** 10.3390/ijms25168719

**Published:** 2024-08-09

**Authors:** Petra Mikulić, Marin Ogorevc, Marin Petričević, Dean Kaličanin, Robert Tafra, Mirna Saraga-Babić, Snježana Mardešić

**Affiliations:** 1 Department of Otorhinolaryngology and Head and Neck Surgery, University Hospital of Split, Spinčićeva 1, 21000 Split, Croatia; psmoje88@gmail.com (P.M.); rtafra2@gmail.com (R.T.); 2 Division of Anatomy, Histology and Embryology, University of Split School of Medicine, Šoltanska 2, 21000 Split, Croatia; marin.ogorevc2@gmail.com (M.O.); smbrakus@gmail.com (S.M.); 3 Institute of Emergency Medicine of Split-Dalmatia County, Spinčićeva 1, 21000 Split, Croatia; marin.petricevic66@gmail.com; 4 Department of Medical Biology, University of Split School of Medicine, Šoltanska 2, 21000 Split, Croatia; dean.kalicanin@mefst.hr

**Keywords:** inner ear, innervation, SOX2, JAGGED1, beta-catenin, vitamin D receptor

## Abstract

Sensorineural hearing loss can be caused by lesions to the inner ear during development. Understanding the events and signaling pathways that drive inner ear formation is crucial for determining the possible causes of congenital hearing loss. We have analyzed the innervation and expression of SOX2, JAGGED1, β-catenin (CTNNB1), and vitamin D receptor (VDR) in the inner ears of human conceptuses aged 5 to 10 weeks after fertilization (W) using immunohistochemistry. The prosensory domains of the human inner ear displayed SOX2 and JAGGED1 expression throughout the analyzed period, with SOX2 expression being more extensive in all the analyzed timepoints. Innervation of vestibular prosensory domains was present at 6 W and extensive at 10 W, while nerve fibers reached the base of the cochlear prosensory domain at 7–8 W. CTNNB1 and VDR expression was mostly membranous and present during all analyzed timepoints in the inner ear, being the strongest in the non-sensory epithelium. Their expression was stronger in the vestibular region compared to the cochlear duct. CTNNB1 and VDR expression displayed opposite expression trends during the analyzed period, but additional studies are needed to elucidate whether they interact during inner ear development.

## 1. Introduction

Hearing loss is one of the most common disabilities and affects approximately 360 million people worldwide [1]. It can be divided into sensorineural, conductive, or mixed. Sensorineural hearing loss is the most common type and can be caused by lesions in the inner ear, cochlear (auditory) nerve, or neurons of the auditory pathway [2]. Congenital hearing loss may stem from environmental factors such as congenital infections, while genetic factors likely predominate in developed countries [3].

Human inner ear development begins at 4 weeks after fertilization (W) with the appearance of otic placodes at the level of the hindbrain [4]. The otic placode invaginates and forms the otocyst, which gives rise to the entire membranous labyrinth of the inner ear. The dorsal part of the otocyst will form the utricle and semicircular ducts, while the ventral part will become the saccule and cochlear duct [5]. The anteromedial region of the otocyst is characterized by SRY-box 2 (SOX2) expression and represents the proneurosensory domain. As neural development begins, prospective neuroblasts from this domain will delaminate and differentiate into bipolar neurons which will form the vestibulocochlear ganglion (VCG) [6]. After neuroblast delamination, the SOX2-positive region is referred to as the prosensory domain and contains precursors for hair cells (HCs), the sensory cells of the inner ear [7]. Specification of the prosensory domain relies on lateral induction mediated by JAGGED1. JAGGED1 induces its own expression in adjacent cells and the expression of SOX2, resulting in the homogenous commitment of otic progenitors to the prosensory fate [8]. Interestingly, there seems to exist an interspecies difference between mice and primates regarding the expression of these factors [9]. While JAGGED1 is expressed early in the mouse inner ear and is essential for SOX2 expression [10], in the common marmoset, SOX2 expression precedes JAGGED1 expression [11]. The relationship between the expression patterns of SOX2 and JAGGED1 has not been described during early human inner ear development. Under the effect of WNT and fibroblast growth factor signaling, cells of the prosensory domain exit the cell cycle and begin expressing Atoh1 and other NOTCH ligands, such as JAGGED2 and DELTA-LIKE 1 [12]. Atoh1 has a key role in HC differentiation, maturation, and survival, while the NOTCH ligands suppress HC fate in adjacent cells through lateral inhibition, resulting in the differentiation of supporting cells [13]. The differentiation of HCs begins between 7 and 8 W in the vestibular maculae [14] and at 8 W in the basal turn of the cochlear duct [15], becoming visible by light microscopy between 10 and 12 W [16]. With the differentiation of HCs, the sensory domains are formed in distinct regions of the membranous labyrinth, three cristae ampullares in the semicircular ducts, two maculae in the utricle and saccule, and the organ of Corti in the cochlear duct [17].

Several investigators showed that VCG neurons send their peripheral processes back to the sensory domains from which they originated from and form synapses with HCs [18,19,20]. Spiral ganglion neurons and their processes are surrounded by peripheral glial cells at 7 W [21]. A light microscopy study described the presence of nerve fibers in the human inner ear epithelium around 4 W [22], but this was refuted by other studies [23,24]. Electron microscopy studies have demonstrated nerve fibers in the macular epithelium at 7 W [25] and penetrating the basal turn of the cochlear duct at 9 W [26,27]. The nerve fibers reach the HCs of the cochlear duct between 11 and 12 W [16] and become abundant by 13 W [28]. The supporting cells secrete ATP, neurotrophin-3, and brain-derived neurotrophic factor, which engage in reciprocal interactions with the HCs and VCG neurons, regulating the formation of synaptic connections [29]. HCs do not require innervation for differentiation and survival as loss of synaptic formation does not alter the structure of inner ear sensory epithelia [30]. Furthermore, the differentiation of epithelial cells in the sensory and non-sensory components of the inner ear occurs simultaneously and is driven by a specific set of genes and transcription factors [31].

WNT signaling is important in the development of the inner ear where it participates in otic specification, the formation of vestibular structures, and the development of the cochlea. The canonical WNT pathway acts through FRIZZLED receptors, with β-catenin (CTNNB1) serving as a major second messenger, culminating in the transcriptional activation of genes [32]. CTNNB1 is normally expressed in the membranes of epithelial cells; however, it can also be ectopically expressed in the cytoplasm and/or nucleus, which marks the activation of canonical WNT signaling [33].

The vitamin D receptor (VDR) is a member of the nuclear receptor superfamily that is highly conserved within vertebrates. It is located predominantly in the cytoplasm; however, upon interacting with the active form of vitamin D, it forms heterodimers with the retinoid X receptor and translocates to the nucleus. After binding with various transcription factors VDR causes up- or downregulation of numerous genes controlled by vitamin D [34,35]. It was described that VDR can upregulate the expression of brain natriuretic peptide which promotes VCG neuron survival and neurite growth in the rat inner ear [36]. Also, loss of VDR expression is associated with early-onset sensorineural hearing loss and vestibular dysfunction in mice [37,38]. Several studies have shown an association between vitamin D deficiency and sensorineural hearing loss and vestibular disorders in humans [39,40,41]. Two studies have even found that treatment outcomes of hearing loss correlate with vitamin D serum levels [42,43]. It is also interesting to note that vitamin D-mediated signaling displays mutual antagonism with canonical WNT signaling in cancers [44]; however, their relationship and expression patterns during inner ear development have not been explored.

While the innervation and role of canonical WNT signaling during mammalian inner ear development have been extensively studied on mouse models, studies examining human inner ear development are sparse, particularly during the earliest stages. The expression of VDR during human inner ear development has not been described to the best of the authors’ knowledge, while animal models demonstrate that VDR deficiency leads to inner ear defects. Therefore, the aim of this study was to analyze the innervation and distribution of SOX2, JAGGED1, CTNNB1, and VDR during early human inner ear development. Additionally, we aimed to explore the relationship between SOX2 and JAGGED1, as well as CTNNB1 and VDR, by comparing their expression patterns throughout the analyzed developmental stages.

## 2. Results

Sections of human conceptuses between 5 and 10 W were analyzed to determine the innervation, SOX2, JAGGED1, CTNNB1, and VDR expression in developing inner ears. The cochlear region (developing cochlear duct) and vestibular apparatus (developing utricle, saccule, and semicircular ducts) were analyzed separately to determine the depth of innervation and protein expression. DAPI staining was used to visualize all cell nuclei.

### 2.1. Morphology of the Developing Human Inner Ear

**Figure 1:**
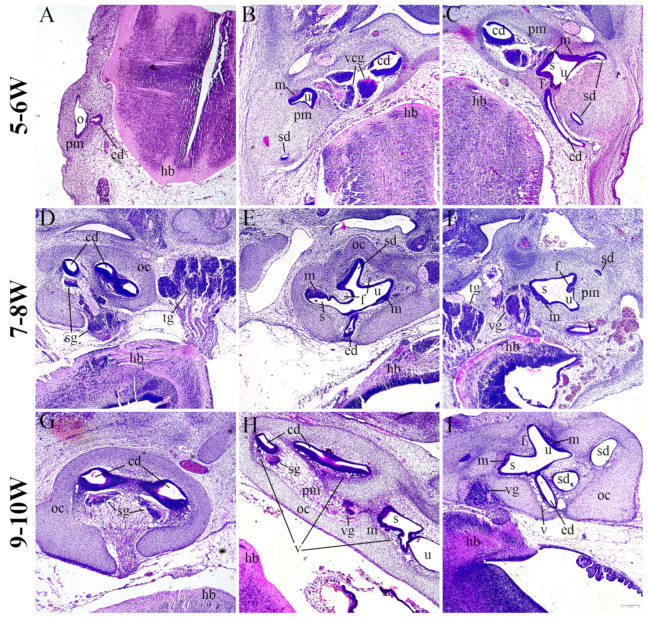
Hematoxylin and eosin staining of the developing human inner ear. Sections through the human inner ear at 5–6 W (**A**–**C**), 7–8 W (**D**–**F**), and 9–10 W (**G**–**I**) display the general morphology at these developmental periods. o—otocyst, pm—periotic mesenchyme, ed—endolymphatic duct, hb—hindbrain, cd—cochlear duct, u—utricle, m—macula, sd—semicircular duct, vcg—vestibulocochlear ganglion, s—saccule, oc—otic capsule, sg—spiral ganglion, tg—trigeminal ganglion, f—epithelial fold, vg—vestibular ganglion, v—vacuolization. The scale bar (bottom right) represents 200 µm.

During the 5th W, the inner ear is in the stage of otic vesicle (otocyst), which shows initial signs of elongation (to form vestibular region dorsally and cochlear region ventrally). The endolymphatic duct extends from the otocyst, approaching the nearby hindbrain which is surrounded by the head mesenchyme (Figure 1A). During further development, the semicircular ducts grow from the utricle and the cochlear duct from the saccule, while the surrounding mesenchyme shows initial signs of chondrification (Figure 1B,C). In the 7th and 8th W, the cochlear duct is coiling and the epithelial cells in its wall will gradually differentiate into the organ of Corti, while the ganglion cells of the 8th nerve form the spiral ganglion. The cartilaginous otic capsule surrounds the cochlear duct (Figure 1D). Further differentiation is also observed in the region of developing semicircular ducts, the junction between the utricle and saccule, and in the developing maculae (Figure 1E,F). In the 9th and 10th W of human inner ear development, the cartilaginous otic capsule is well defined, and contains the coiled cochlear duct and spiral ganglion cells which approach the ventral wall of cochlear duct (Figure 1G). The cochlear duct is continuous with the nearby saccule (Figure 1H). Communication of the utricle and saccule, with mutually perpendicular maculae and semicircular ducts close to the utricle are also enclosed by a cartilaginous shell. Vacuolization is observed in the surrounding mesenchyme, which will gradually develop into the perilymphatic space (Figure 1I).

### 2.2. SOX2 and JAGGED1 Expression Characterizes Prosensory Domains of the Developing Human Inner Ear

**Figure 2:**
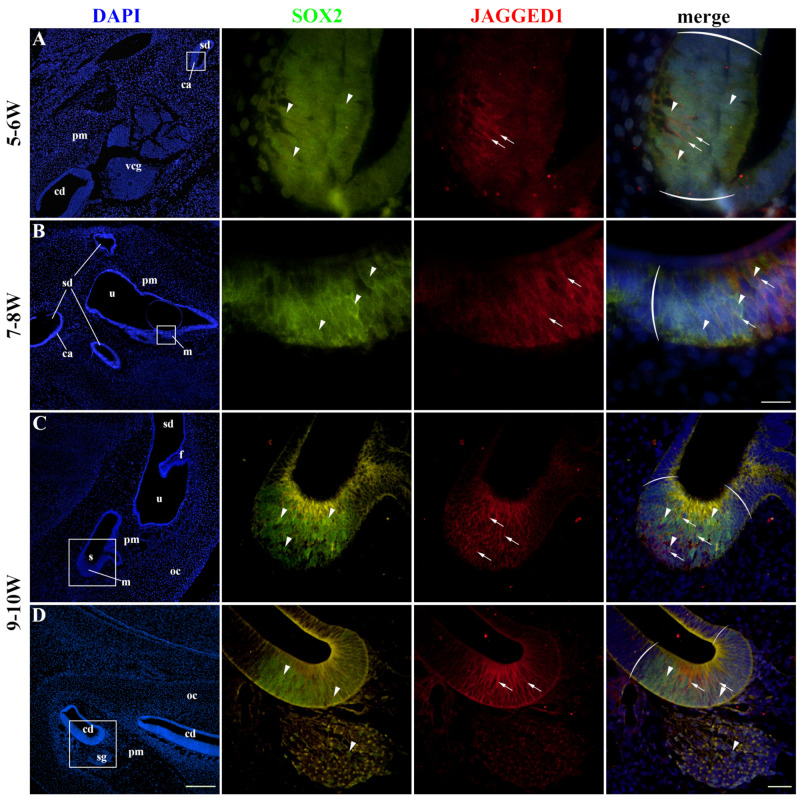
SOX2 and JAGGED1 expression in the developing human inner ear. The co-expression of SOX2 and JAGGED1 was analyzed at 5–6 W (**A**), 7–8 W (**B**) and 9–10 W (**C**,**D**). cd—cochlear duct, u—utricle, m—macula, ca—crista ampullaris, vcg—vestibulocochlear ganglion, pm—periotic mesenchyme, sd—semicircular duct, s—saccule, oc—otic capsule, f—epithelial fold, sg—spiral ganglion. Nuclear SOX2 expression is visible in prosensory domains and some sg cells (arrowheads), while JAGGED1 expression is limited to the prosensory domains (arrows). Round brackets mark the border of the prosensory region. First column ×100 magnification, scale bar 500 µm; other columns ×1000 magnification, scale bar 50 µm in the first two rows; ×400 magnification, scale bar 100 µm in the last two rows.

SOX2 expression characterized prosensory domains in the developing cochlear duct and vestibular apparatus, which will eventually become the organ of Corti, maculae, and cristae ampullares. SOX2 expression was predominately nuclear, with some cells displaying intense cytoplasmic staining. JAGGED1 membranous expression was co-localized with SOX2 expression in the developing inner ear at 5–6 W (Figure 2A). The expression pattern of SOX2 was more extensive compared to JAGGED1. The same pattern was observed at 7–8 W (Figure 2B) and 9–10 W (Figure 2C,D), with SOX2 expression being consistently more extensive. The membranous staining pattern of JAGGED1 was present in both vestibular and cochlear prosensory regions of the developing inner ear, without visible significant differences (Figure 2C,D). Some cells of the spiral ganglion also displayed SOX2 nuclear staining; however, they were negative for JAGGED1 (Figure 2D). These cells likely represent developing glial (satellite) cells.

### 2.3. Innervation of the Developing Human Inner Ear

**Figure 3:**
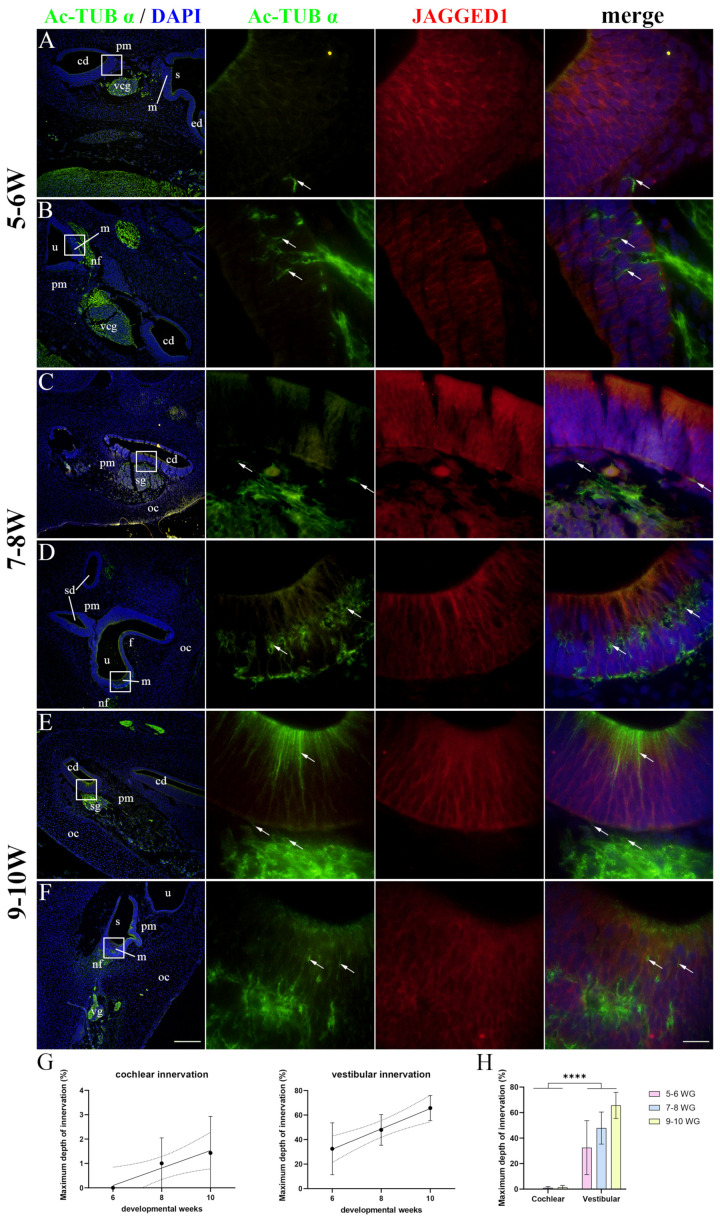
Innervation of the developing human inner ear. The cochlear (**A**,**C**,**E**) and vestibular (**B**,**D**,**F**) regions of the human inner ear at 5–6 W (**A**,**B**), 7–8 W (**C**,**D**), and 9–10 W (**E**,**F**) are displayed. cd—cochlear duct, u—utricle, m—macula, vcg—vestibulocochlear ganglion, pm—periotic mes-enchyme, sd—semicircular duct, s—saccule, oc—otic capsule, f—epithelial fold, sg—spiral ganglion, nf—nerve fibers, ed—endolymphatic duct, vg—vestibular ganglion. Acetylated-α-tubulin (arrows) marks nerve fibers, while JAGGED1 expression characterizes prosensory domains. First column ×100 magnification, scale bar 500 µm; other columns ×1000 magnification, scale bar 50 µm. The dynamics of the innervation of the cochlear and vestibular epithelium are displayed by linear regression modeling of maximum depth of innervation during 5–10 W (**G**). The maximum depth of innervation (**H**) of cochlear and vestibular prosensory regions was analyzed by two-way ANOVA and Tukey’s multiple comparisons test. The data are presented as means with SD (vertical error bars). **** *p* < 0.0001.

Acetylated-α-tubulin (Ac-TUB α) was used to display nerve fibers as it is only present in structures with stabilized microtubules, such as axons, while JAGGED1 marked the prosensory domains of the developing cochlear duct and vestibular apparatus. At 5–6 W, no nerve fibers were present in the cochlear duct (Figure 3A), while the prospective maculae of the vestibular apparatus had some nerve fibers present in the basal half of the epithelium (Figure 3B). At 8 W, the nerve fibers had reached the cochlear duct epithelium (Figure 3C). At the same timepoint, the developing maculae were more densely innervated, and the nerve fibers reached the basal surfaces of differentiating HCs (Figure 3D). At 9–10 W, the developing cochlear duct still had nerve fibers present mostly at the basal surface of the epithelium (Figure 3E), while the vestibular maculae were densely innervated with nerve fibers surrounding the HCs (Figure 3F). Strong Ac-TUB-α immunoexpression was also observed in the apical region of the cochlear duct prosensory domain at 9–10 W, probably representing the processes of developing phalangeal and pillar cells (Figure 3E). When analyzing the maximum depth of innervation, a significant linear trend among developmental periods was found for both the cochlear (R^2^ = 26.03%, β = 0.358 ± 0.290) and vestibular (R^2^ = 47.40%, β = 8.294 ± 4.196) prosensory domains (Figure 3G). The innervation of the vestibular apparatus was more extensive (*p* < 0.0001) compared to the cochlear duct throughout the entire analyzed period (Figure 3H).

### 2.4. CTNNB1 Expression in the Developing Human Inner Ear

**Figure 4:**
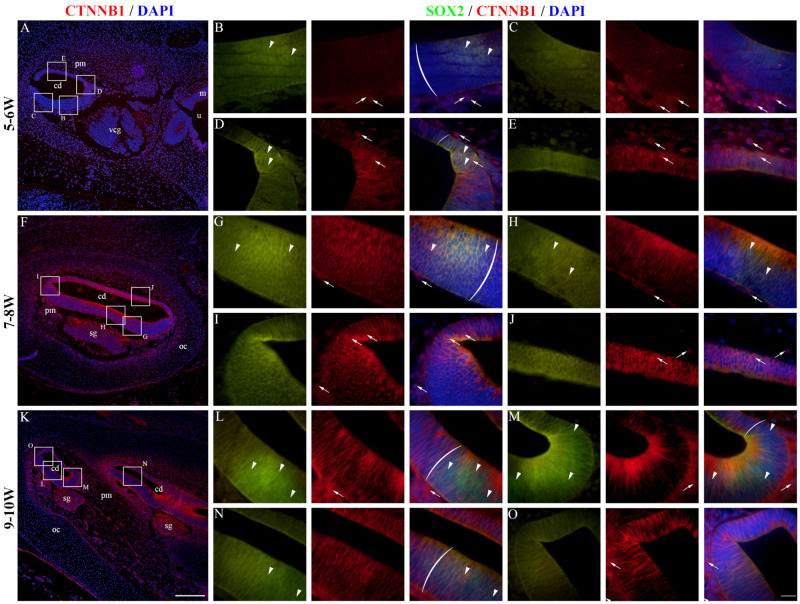
CTNNB1 expression in the developing cochlear duct. The cochlear duct of the human inner ear at 5–6 W (**A**–**E**), 7–8 W (**F**–**J**), and 9–10 W (**K**–**O**) is displayed. cd—cochlear duct, u—utricle, m—macula, vcg—vestibulocochlear ganglion, pm—periotic mesenchyme, oc—otic capsule, sg—spiral ganglion. Ectopic CTNNB1 expression is marked by arrows, while nuclear SOX2 expression is marked by arrowheads. Round brackets mark the border of the prosensory region. First column ×100 magnification, scale bar 500 µm; other columns ×1000 magnification, scale bar 50 µm.

**Figure 5:**
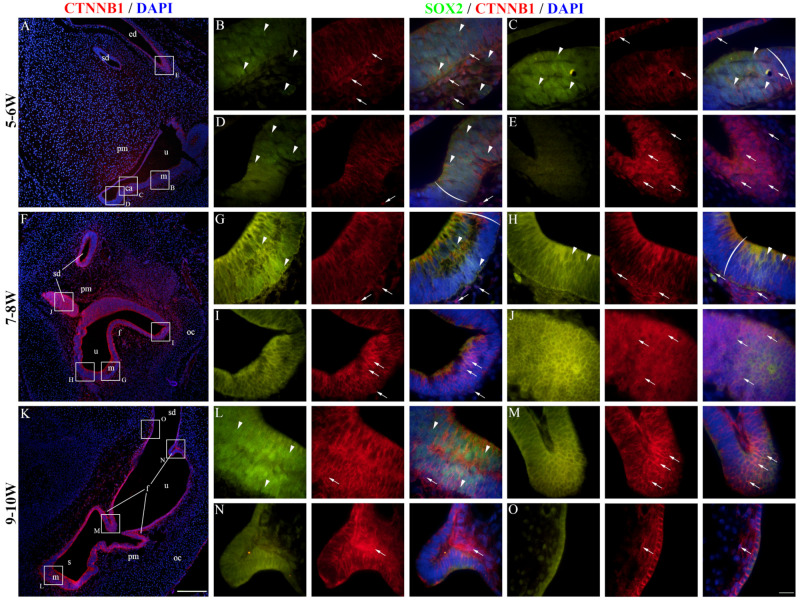
CTNNB1 expression in the developing vestibular apparatus. The vestibular apparatus of the human inner ear at 5–6 W (**A**–**E**), 7–8 W (**F**–**J**), and 9–10 W (**K**–**O**) is displayed. u—utricle, m—macula, ca—crista ampullaris, pm—periotic mesenchyme, sd—semicircular duct, s—saccule, oc—otic capsule, f—epithelial fold, ed—endolymphatic duct. Ectopic CTNNB1 expression is marked by arrows, while nuclear SOX2 expression is marked by arrowheads. Round brackets mark the border of the prosensory region. First column ×100 magnification, scale bar 500 µm; other columns ×1000 magnification, scale bar 50 µm.

**Figure 6:**
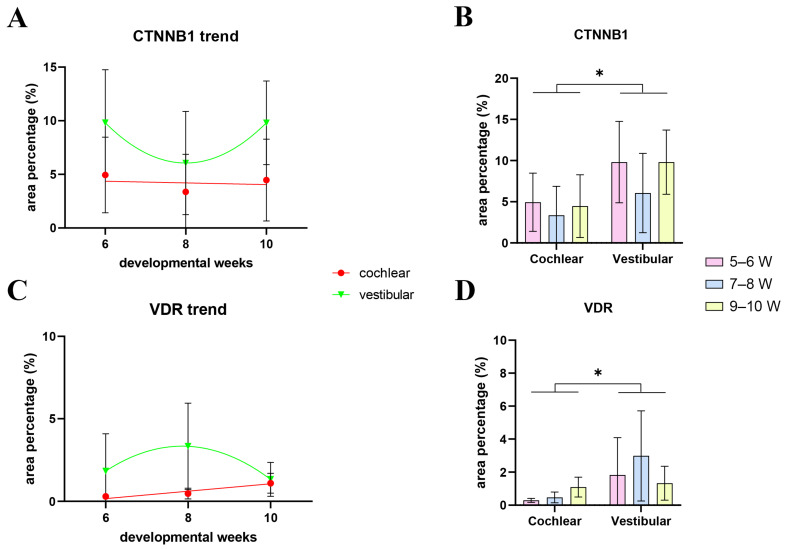
Analyses of CTNNB1 and VDR in the developing human inner ear. The changes in expression of CTNNB1 (**A**) and VDR (**C**) are demonstrated by linear and quadratic regression modeling of area percentages through developmental periods (5–10 W) in the cochlear and vestibular regions of the developing human inner ear. The area percentages of CTNNB1 (**B**) and VDR (**D**) in the cochlear and vestibular regions of the developing human inner ear were analyzed by two-way ANOVA and Tukey’s multiple comparisons test. The data are presented as means with SD (vertical error bars). * *p* < 0.05.

At 5–6 W, CTNNB1 displayed a mostly membranous staining pattern in the epithelium of the developing cochlear duct, meaning that canonical WNT signaling is not active in the majority of cochlear duct cells at this stage (Figure 4A). The cochlear floor region displayed little to no CTNNB1 staining in the SOX2-positive area (Figure 4B), with more apparent membranous staining laterally near the outer sulcus (Figure 4C). Nuclear CTNNB1 staining was visible in the SOX2-positive area on the medial border of the floor region (Figure 4D) and in the epithelium of the roof region (Figure 4E), indicating active canonical WNT signaling in these regions. At 7–8 W, the expression pattern was similar to 5–6 W (Figure 4F). The floor regions containing the SOX2-positive prosensory domain displayed mostly membranous CTNNB1 staining (Figure 4G,H), while nuclear CTNNB1 staining and active canonical WNT signaling characterized the epithelial cells of the roof region and the border between the two regions (Figure 4I,J). At 9–10 W, most cells of the cochlear duct had a membranous CTNNB1 staining pattern, indicating that canonical WNT signaling is not active (Figure 4K–O). The vestibular regions at 5–6 W contained both cells displaying membranous and nuclear CTNNB1 expression (Figure 5A). The maculae and developing cristae ampullares (SOX2-positive) displayed membranous staining (Figure 5B–D), while nuclear expression could be seen in the transitional and dark cell epithelium (Figure 5C), as well as in the endolymphatic duct (Figure 5E), indicating that canonical WNT signaling is active in these regions. A similar pattern was seen at 7–8 W (Figure 5F), with membranous CTNNB1 staining present in the maculae (Figure 5G,H) and nuclear expression in the non-sensory area near the epithelial fold (Figure 5I) and in the semicircular ducts (Figure 5J). Strong membranous CTNNB1 staining was visible at 9–10 W (Figure 5K), especially in the maculae (Figure 5L) and dark cell epithelium (Figure 5O). Nuclear CTNNB1 expression was present in some but not all the epithelial folds (Figure 5M,N). Many cells of the periotic mesenchyme around the cochlear duct and vestibular apparatus showed nuclear CTNNB1 expression in all the observed developmental periods (Figure 4 and Figure 5). Therefore, active canonical WNT signaling is mostly present in the non-sensory areas of the inner ear epithelium and the periotic mesenchyme, while the SOX2-positive prosensory domains show no active canonical WNT signaling during early development. Analysis of the area percentage of CTNNB1 expression did not reveal a significant linear trend among developmental periods in the cochlear duct (R2 = 0.12%, β = −0.078 ± 0.859), while the expression in the vestibular epithelium showed a quadratic trend, with a nadir in expression at 7–8 W (R^2^ = 11.15%) (Figure 6A). The vestibular regions displayed significantly higher CTNNB1 expression than the developing cochlear duct (*p* = 0.0151) when analyzing across all observed timepoints (Figure 6B).

### 2.5. VDR Expression in the Developing Human Inner Ear

**Figure 7:**
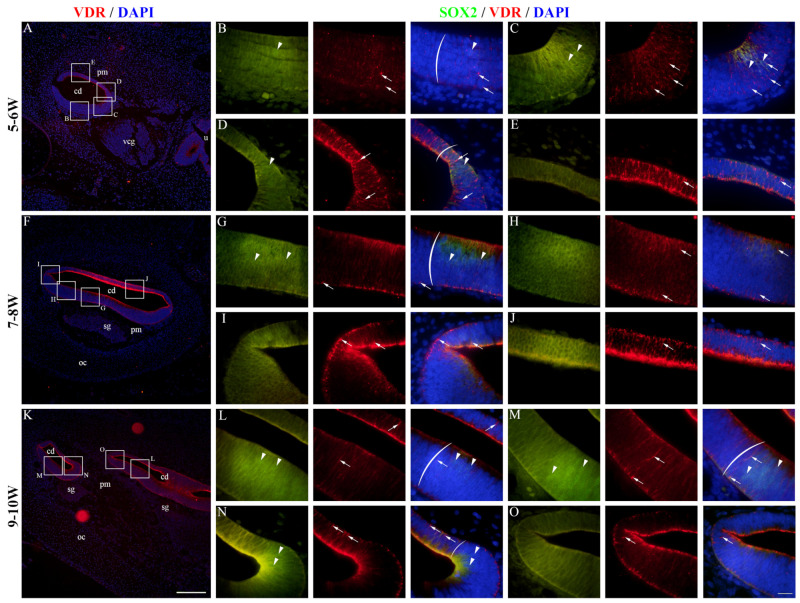
VDR expression in the developing cochlear duct. The cochlear duct of the human inner ear at 5–6 W (**A**–**E**), 7–8 W (**F**–**J**), and 9–10 W (**K**–**O**) is displayed. cd—cochlear duct, u—utricle, vcg—vestibulocochlear ganglion, pm—periotic mesenchyme, oc—otic capsule, sg—spiral ganglion. Nuclear VDR expression is marked by arrows, while nuclear SOX2 expression is marked by arrowheads. Round brackets mark the border of the prosensory region. First column ×100 magnification, scale bar 500 µm; other columns ×1000 magnification, scale bar 50 µm.

**Figure 8:**
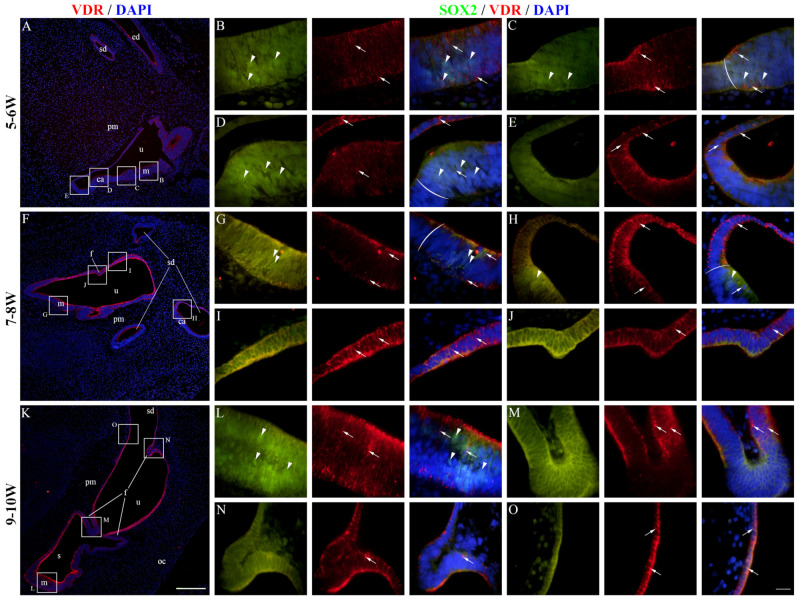
VDR expression in the developing vestibular apparatus. The vestibular apparatus of the human inner ear at 5–6 W (**A**–**E**), 7–8 W (**F**–**J**), and 9–10 W (**K**–**O**) is displayed. u—utricle, m—macula, ca—crista ampullaris, pm—periotic mesenchyme, sd—semicircular duct, s—saccule, oc—otic capsule, f—epithelial fold, ed—endolymphatic duct. Nuclear VDR expression is marked by arrows, while nuclear SOX2 expression is marked by arrowheads. Round brackets mark the border of the prosensory region. First column ×100 magnification, scale bar 500 µm; other columns ×1000 magnification, scale bar 50 µm.

At 5–6 W, VDR expression was present in the epithelium of the developing cochlear duct (Figure 7A). The cochlear floor region displayed mostly membranous/cytoplasmatic VDR staining, while nuclear staining could be seen in some SOX2-positive and SOX2-negative cells (Figure 7B,C). VDR expression in the floor region was higher near the border with the roof region (Figure 7C,D), and the highest expression was observed in the roof region (Figure 7E). At 7–8 W, the expression pattern was similar to 5–6 W (Figure 7F), with the floor regions having some cells with nuclear VDR staining, both within and outside the prosensory domain (Figure 7G,H). The expression of VDR was generally higher in the roof regions (Figure 7I,J). The same expression pattern was present at 9–10 W (Figure 7K–O). The vestibular regions at 5–6 W had both membranous/cytoplasmatic and nuclear VDR expression (Figure 8A). The cells of the maculae and developing cristae ampullares (SOX2-positive) displayed both staining patterns (Figure 8B–D), and the same was true for the non-sensory transitional and dark cell epithelium areas (Figure 8D,E). A similar pattern was seen at 7–8 W (Figure 8F) with VDR staining being stronger in the non-sensory dark cell epithelium compared to the prosensory domains (Figure 8G–I), with the exception of a forming epithelial fold that showed weaker VDR expression (Figure 8J). Strong VDR staining was visible at 9–10 W in the dark cell epithelium (Figure 8K). Nuclear VDR expression was present in the maculae (Figure 8L) and some cells of the epithelial folds, while most cells of the folds were devoid of VDR expression (Figure 8M,N). Nuclear VDR staining was present in the dark cell epithelium (Figure 8O). VDR staining was seldom present in cells of the periotic mesenchyme, mostly at 5–6 W, while a majority of the cells showed no VDR expression (Figure 7 and Figure 8). Analysis of the area percentage of VDR expression showed a significant linear trend among developmental periods in the cochlear duct (R^2^ = 32.79%, β = 0.224 ± 0.120), while the expression in the vestibular epithelium showed a quadratic trend, with a peak in expression at 7–8 W (R^2^ = 12.46%) (Figure 6C). The vestibular region had significantly higher VDR expression than the developing cochlear duct (*p* = 0.0348) when analyzing across all observed timepoints (Figure 6D).

## 3. Discussion

Our study focused on the innervation of the prosensory domains, as well as the expression of Sox2, JAGGED1, CTNNB1, and VDR in the developing human inner ear. We have analyzed samples from conceptuses aged 5 to 10 W in order to determine when innervation begins and how it proceeds during early development. We have also described the spatiotemporal expression pattern and mutual relationship of SOX2, JAGGED1, CTNNB1, and VDR.

In our samples, SOX2 and JAGGED1 had similar expression patterns in the cells of the developing inner ear as were described in other studies [7,8,45]. These cells belong to the prosensory domain from which HCs will develop. Although SOX2 is a transcription factor, its expression in the cytoplasm has been described in both embryonic and cancer stem cells [46,47]. Acetylation of SOX2 has been described as a post-translational regulatory mechanism that causes nuclear export, accumulation in the cytoplasm, and subsequent proteosomal degradation, leading to a reduction in transcription of its target genes [46]. Given the cytoplasmic expression of SOX2 found in our study, this mechanism might be involved in SOX2 patterning during inner ear development; however, experimental studies are needed to ascertain this. Even though it was previously described that JAGGED1 induces SOX2 expression via lateral induction [8], the expression of SOX2 in our samples was more extensive than that of JAGGED1, suggesting that SOX2 expression is also under the control of other factors. This is in line with studies on primate models which have demonstrated that SOX2 expression precedes JAGGED1 expression during inner ear development [9,11]. Given the differences between murine and primate models for prosensory domain specification [9,10,11], our results indicate that primate models more accurately represent the early stages of inner ear prosensory domain specification. It is important to note that, unlike Hosoya et al. [11], we have not found a developmental stage when SOX2 expression is present and JAGGED1 expression is absent. Therefore, investigation of earlier stages of human inner ear development, if possible, is necessary to better elucidate the process of prosensory domain specification and the relationship between SOX2 and JAGGED1.

When observing the innervation of the prosensory domains, we found that the nerve fibers first reach the developing cochlear duct at 8 W and remain mostly at the basal surface at 9–10 W, which is in line with multiple studies [26,27,31]. The vestibular region (developing maculae) was already visibly innervated at 6 W. Chacko et al. have described the earliest innervation of human vestibular structures at 8 W [48], while Sans and Dechesne have found nerve fibers in the macular epithelium at 7 W; however, their studies did not analyze any conceptuses at earlier developmental periods [25]. The innervation of vestibular prosensory domains became more excessive at later developmental periods and nerve fibers reached the most differentiating HCs at 9–10 W, which is in line with the aforementioned study [48]. Interestingly, even though SOX2 expression was more widespread than JAGGED1, the nerve fibers penetrated the prosensory domain epithelium only in the JAGGED1-positive region, suggesting that JAGGED1 expression is associated with axon guidance.

Membranous CTNNB1 expression was present in the human inner ear epithelium throughout all the observed periods, becoming the strongest at 9–10 W. Nuclear CTNNB1 expression was mostly seen in the non-sensory regions and surrounding mesenchyme. Nuclear CTNNB1 expression usually indicates the activation of canonical WNT signaling, which has been shown to induce the proliferation and differentiation of epithelial cells of the developing murine cochlear duct [33,45,49,50]. It was also described that CTNNB1 may play a role in the proliferation of the vestibular epithelium in rats [51]. It has been shown that CTNNB1 expression is lost after the formation of the otocyst from the otic cup in rats [52]; however, CTNNB1 expression was present in the 5 W otocyst of our samples. Further investigation is needed to determine whether CTNNB1 expression is lost and then regained during human otocyst formation or if the expression is present throughout the entire process of development.

The role of VDR in inner ear development and function has not been extensively studied. It has been described that downregulation of VDR signaling is associated with benign paroxysmal positional vertigo, sensorineural hearing loss, and other inner ear disorders in humans [39,40,41,53]. A study on zebrafish found that the knockdown of VDR resulted in malformed vestibular structures and decreased HC production [54]. Zou et al. found that knockout of VDR in mice had no effects on cochlear morphology but resulted in early-onset sensorineural hearing loss associated with reduced apoptosis [38]. We found that VDR is expressed in the human inner ear during early development, especially in the vestibular regions, which corresponds to the findings of the previously mentioned studies that loss of VDR results in vestibular but not cochlear malformations [38,54]. Considering that the expression was mostly membranous or cytoplasmatic, and seldom nuclear, we presume that vitamin D affects the developing human inner ear mostly through non-genomic pathways [55]. Studies on VDR expression in the human inner ear at later stages of development and postnatally are needed to further elucidate the distribution and possible role of VDR for inner ear function.

When analyzing the relationship between CTNNB1 and VDR expression in the developing inner ear, we have discovered that nuclear CTNNB1 expression and VDR expression display opposite patterns when observing the same regions (Supplementary Figure S2). This relationship between nuclear CTNNB1 expression and VDR has been previously described as mutually antagonistic in several studies [44,56,57]. Considering that nuclear CTNNB1 expression is associated with proliferation and VDR signaling with apoptosis in the inner ear [38,45], we presume that these factors are needed to properly regulate the fine balance between proliferation and apoptosis, which is necessary for normal human inner ear development [58].

The main limitations of our study are the small sample size and the fact that we cannot perform quantitative studies of protein expression such as Western blotting or flow cytometry since our samples are archived sections of embryonic/fetal tissue that was formalin-fixed and paraffin-embedded. An additional limitation is that immunofluorescent staining can have varying results depending on the experimental conditions, and that non-specific staining is often present and can interfere with the interpretation of results. With this in mind, we consider the results of our study to be valuable as we provide insight about the innervation as well as SOX2, JAGGED1, CTNNB1, and VDR expression in the human inner ear at the earliest stages of development. Additional experimental and observational studies on animal models are needed to better understand the interplays of these factors during inner ear development.

## 4. Materials and Methods

### 4.1. Human Samples

In our study, we investigated a total of 12 human developing inner ear samples which belong to the archive collection of human embryos and fetuses in the Department of Anatomy, Histology, and Embryology, at the University of Split School of Medicine. The embryonic and fetal tissue specimens were obtained after spontaneous abortions or tubal pregnancies, with the permission of the Ethical and Drug Committee of the University Hospital in Split in accordance with the Helsinki Declaration (class: 003-08/16-03/0001, approval number: 2181-198-03-04-16-0024), and only conceptuses with no signs of maceration, abnormalities, or morphological changes were included in the study. The ages of conceptuses, stated as weeks after fertilization (W), were estimated by crown–rump length and menstrual data and correlated with the Carnegie stages. Each developmental age group (5–6 W, 7–8 W, and 9–10 W) contained four conceptuses.

### 4.2. Immunofluorescence Staining

Following the fixation in 4% paraformaldehyde in phosphate buffer saline (PBS), tissue was dehydrated in graded ethanol solutions. The tissue was then embedded in paraffin blocks, cut serially as 5 µm thick sections, and mounted on glass slides. Every tenth section was stained with hematoxylin and eosin to describe the stages in normal inner ear development and to confirm the preservation of tissue morphology. The immunofluorescence staining protocol was performed as described previously [59]. Briefly, the tissue slides were deparaffinized in xylol and rehydrated in graded ethanol solutions. The samples were then heated in a sodium citrate buffer (pH 6.0) using a steam cooker for 30 min. After a round of washing in phosphate-buffered saline (PBS), protein blocking buffer (Protein Block ab64226, Abcam, Cambridge, UK) was applied for 20 min in a humid chamber. Combinations of primary antibodies (Table 1) were applied and incubated overnight in the humid chamber. The primary antibodies were rinsed by washing the samples in PBS, and appropriate secondary antibodies (Table 1) were applied for 1 h in the humid chamber. Afterward, samples were once again washed in PBS, counterstained with 4′,6-diamidino-2-phenylindole (DAPI) for 2 min, and cover-slipped using a mounting medium (ImmuMount, Thermo Shandon, Pittsburgh, PA, USA). The specificity of staining was controlled by omitting primary or secondary antibodies from the staining protocol and capturing images using identical microscope settings (Supplementary Figure S1). The stained samples were analyzed using an epifluorescence microscope (Olympus BX61, Tokyo, Japan), and images were captured with a mounted digital camera (Nikon Ri-D2, Nikon, Tokyo, Japan) using NIS-Elements F software version 3.0 (Nikon, Tokyo, Japan).

### 4.3. Innervation Analysis

The innervation of prosensory domains was estimated by determining the maximum depth of nerve fiber penetration into the inner ear epithelium. It was calculated as the ratio of the intraepithelial length of the farthest-reaching nerve fiber and the epithelial thickness at the same point.

### 4.4. Immunofluorescence Signal Quantification

In order to quantify the immunofluorescence signal of the analyzed proteins, we calculated the area percentage that the signal took up in the captured images as described previously [60]. Briefly, for each sample, we captured images of the developing inner ear epithelium using a 40× objective lens. Each image underwent identical processing steps. Using the Adobe Photoshop version 21.0.2 (Adobe, San Jose, CA, USA) Lasso tool, the regions of interest were selected and extracted from the images. Afterwards, the fluorescent signal of the analyzed protein was isolated with the median filter and triangle thresholding methods in ImageJ software version 1.53o (NIH, Bethesda, MD, USA). The area percentage of the isolated signal was calculated and corrected relative to the original image from which it was extracted. The figures displayed were assembled in Adobe Photoshop after subtracting the background and slightly enhancing the contrast of the original captured images.

### 4.5. Statistical Analysis

Statistical analysis was performed using GraphPad Prism version 9.0.0 software (GraphPad Software, San Diego, CA, USA). All results are presented as the mean and standard deviation of the calculated percentages. The normality of distribution of the data was determined using the Shapiro–Wilk test. Two-way analysis of variance (ANOVA) with Tukey’s post hoc test was used to determine the statistical significance of the difference in protein expression and maximum depth of nerve fiber penetration between the analyzed groups of samples. Analyses of trends for innervation and expression of CTNNB1 and VDR were performed by regression modeling. Linear trends were described by the slope (β) of the regression line. The goodness of fit measure used was the determination coefficient (R^2^). Statistical significance was set at *p* < 0.05.

## 5. Conclusions

SOX2 and JAGGED1 are expressed in the prosensory domains of the human inner ear between 5–10 W, with SOX2 expression being consistently more extensive. Nerve fibers can be seen penetrating the vestibular maculae of the human inner ear at 6 W and at the base of the cochlear duct at 8 W. CTNNB1 and VDR are expressed throughout the inner ear epithelium during early development, with non-sensory and vestibular regions demonstrating the strongest expression.

## Figures and Tables

**Table 1:** Antibodies used in the study.

	Antibodies	Host	Code No.	Dilution	Source
Primary	Anti-Acetylated-alpha-tubulin	Mouse	12152	1:500	Cell Signaling Technology (CST), (Danvers, MA, USA)
	Anti-Beta-catenin	Mouse	2677	1:200	Cell Signaling Technology (CST), (Danvers, MA, USA)
	Anti-Vitamin D Receptor	Mouse	sc-13133	1:50	Santa Cruz Biotechnology, Dallas, TX, USA
	Anti-JAGGED1	Goat	AF1277	1:100	R&D Systems, Minneapolis, MN, USA
	Anti-SOX2	Rabbit	3579	1:400	Cell Signaling Technology (CST), (Danvers, MA, USA)
Secondary	Alexa Fluor^®^488 Anti-Mouse lgG	Donkey	715-545-150	1:400	Jackson Immuno Research Laboratories, Inc., Baltimore, PA, USA
	Alexa Fluor^®^488 Anti-Rabbit lgG	Donkey	711-545-152	1:400	Jackson Immuno Research Laboratories, Inc., Baltimore, PA, USA
	Rhodamine Red™-X Anti-Mouse IgG	Donkey	715-295-151	1:400	Jackson Immuno Research Laboratories, Inc., Baltimore, PA, USA
	Rhodamine Red™-X Anti-Goat IgG	Donkey	705-295-003	1:400	Jackson Immuno Research Laboratories, Inc., Baltimore, PA, USA

## Data Availability

The data presented in this study are available on request from the corresponding author.

## Supplementary Materials

The following supporting information can be downloaded at: https://www.mdpi.com/article/10.3390/ijms25168719/s1.
