# Supplementary material for: SOX2, JAGGED1, β-Catenin, and Vitamin D Receptor Expression Patterns during Early Development and Innervation of the Human Inner Ear
Source: Int J Mol Sci. 2024 Aug 9;25(16):8719. doi: 10.3390/ijms25168719 (PMC11354891; doi:10.3390/ijms25168719)
Supplement: Supplementary file 1 [file ijms-25-08719-s001.zip › ijms-3115127-supplementary.pdf]

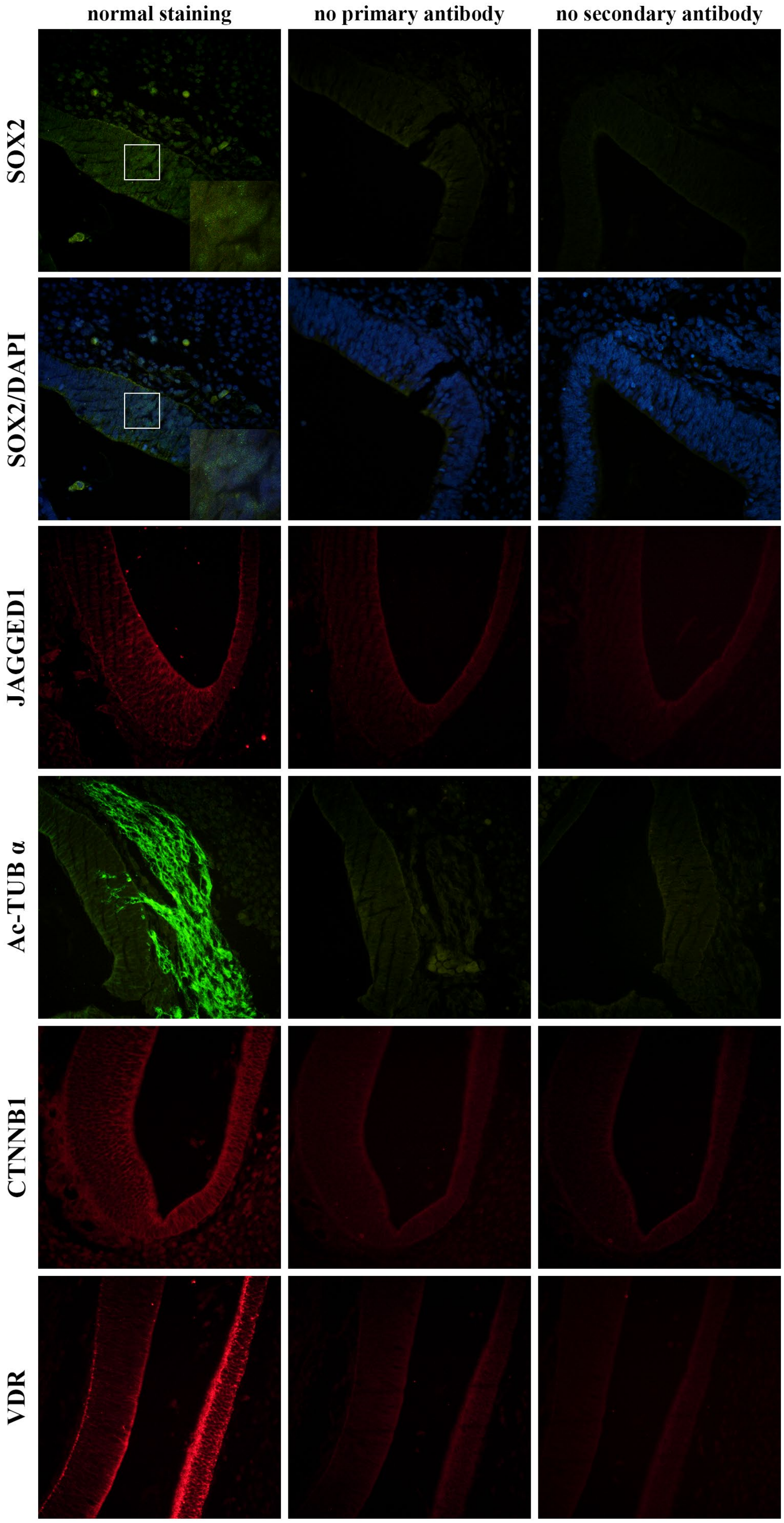

Figure S1. Negative controls for staining.

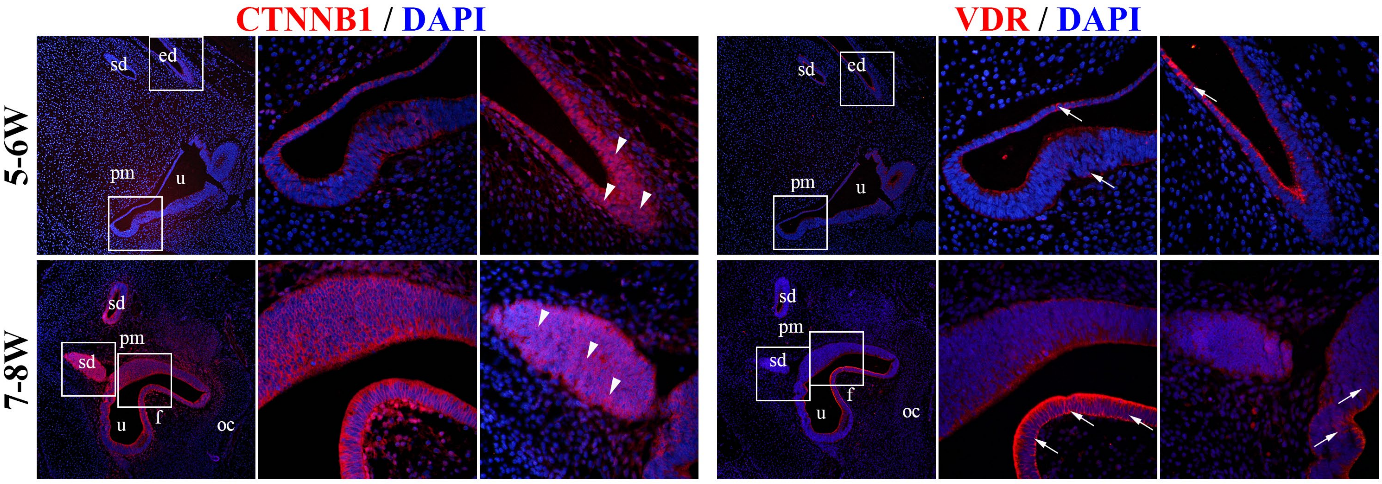

Figure S2. Comparison of CTNNB1 and VDR expression in the developing human inner ear.
